# *Chlamydia trachomatis*-Specific Antibodies and In Vitro Fertilization Outcome

**DOI:** 10.3390/biomedicines13082032

**Published:** 2025-08-20

**Authors:** Natasa Djordjevic, Ana Todorovic, Predrag Sazdanovic, Maja Sazdanovic, Marija Sorak, Aleksandra Gavrilovic, Aida Parandilovic, Eliana Garalejic, Marija Vujovic, Sanja Matic, Suzana Popovic, Dejan Baskic

**Affiliations:** 1 Department of Pharmacology and Toxicology, Faculty of Medical Sciences, University of Kragujevac, 34000 Kragujevac, Serbia; natashadj2002@yahoo.com; 2 Centre for Microbiology, Institute of Public Health Kragujevac, 34000 Kragujevac, Serbia; ana.todorovic.nani@gmail.com (A.T.); dejan.baskic@gmail.com (D.B.); 3 Department of Anatomy, Faculty of Medical Sciences, University of Kragujevac, 34000 Kragujevac, Serbia; 4 Centre of IVF, University Clinical Centre Kragujevac, 34000 Kragujevac, Serbia; sorakmarijakg@gmail.com (M.S.); alexstojanovic2207@gmail.com (A.G.); aidica85@gmail.com (A.P.); 5 Department of Histology and Embryology, Faculty of Medical Sciences, University of Kragujevac, 34000 Kragujevac, Serbia; sazdanovicm@gmail.com; 6 Department for Gynecology and Obstetrics, Faculty of Medical Sciences, University of Kragujevac, 34000 Kragujevac, Serbia; 7 Clinic for Gynecology and Obstetrics “Narodni Front”, Faculty of Medicine, University of Belgrade, 11000 Belgrade, Serbia; elag@sezampro.rs (E.G.); mvujovic67@gmail.com (M.V.); 8 Faculty of Medical Sciences, University of Kragujevac, 34000 Kragujevac, Serbia; sanjad.matic@gmail.com; 9 Centre for Molecular Medicine and Stem Cell Research, Department of Pharmacy, Faculty of Medical Sciences, University of Kragujevac, 34000 Kragujevac, Serbia; popovic007@yahoo.com

**Keywords:** *Chlamydia trachomatis*, IgG, IgA, age, IVF

## Abstract

**Objectives**: *Chlamydia trachomatis* (CT) infection affects female fertility. The purpose of our study was to assess the association between serological and follicular fluid markers of CT infection and in vitro fertilization (IVF) success. **Methods**: This prospective multicenter cohort study included female patients undergoing an IVF procedure in Serbia. The IVF procedure was performed according to the standard protocol. Serum and follicular fluid samples were collected during IVF, and anti-major outer membrane protein (anti-MOMP) IgG and IgA were determined by the Enzyme-Linked Immunosorbent Assay (ELISA) test. **Results**: A significantly higher embryo implantation rate was detected among patients negative for antibodies in follicular fluid (OR (95% CI): 5.254 (1.055; 26.152)). There was a trend toward increased risk of IVF failure in patients positive for either IgG or IgA in follicular fluid, or positive for IgG in serum. Older age was associated with lower odds for successful implantation (OR (95% CI): 0.888 (0.820; 0.962)), biochemical pregnancy (OR (95% CI): 0.890 (0.817; 0.969)), and live birth (OR (95% CI): 0.906 (0.833; 0.985)). **Conclusions**: Our results suggest that the presence of chlamydial anti-MOMP IgG and IgA in the serum and follicular fluid of infertile women could be indicative of lower IVF success rate, and that advanced maternal age is associated with higher risk of IVF failure.

## 1. Introduction

Infertility has been defined as a disease of the reproductive system, presented by failure to establish clinical pregnancy after one year of regular trying [1]. Affecting approximately 17.5% of the adult world population [2], it represents a significant medical, social, and psychological burden at an individual level and a major ongoing challenge for public health globally. Infertility affects both sexes, yet in women the problem is usually more complex, and often further aggravated by treatment invasiveness and its health-, economic, and career-related complications, as well as by frequent stigmatization and victimization in parenthood-oriented societies [3,4].

There are many causes of female infertility, such as older age, ovulatory disorders, and uterine and tubal abnormalities [5,6,7,8,9,10], but the main preventable ones belong to sexually transmitted infections [3,11,12]. Among more than 30 different pathogens that are sexually transmissible [11], the most common bacteria worldwide is *Chlamydia trachomatis* (CT) [13]. This infection usually displays an asymptomatic clinical course [14] that often hinders timely diagnosis, causing detrimental treatment delay: up to 45% of women with an untreated CT infection develop complications such as pelvic inflammatory disease (PID) or tubal factor infertility (TFI), which dramatically increase the risk of both infertility and ectopic pregnancy [15,16,17,18,19]. To combat female infertility, different strategies have been developed, including lifestyle changes, controlled ovarian hyperstimulation with or without intrauterine insemination, and operative hysteroscopy, but in vitro fertilization (IVF) has been associated with the highest live birth rates as compared to other methods [5,20].

IVF represents the most successful method of assisted reproductive technology [5], which employs standard protocols for controlled ovarian stimulation, oocyte aspiration, insemination, embryo culture, and transcervical replacement of embryos, with an aim to achieve pregnancy [20]. Unfortunately, the overall IVF success rate, expressed as pregnancy and delivery rate per aspiration (27.0% and 19.5%, respectively [21]), suggests that most IVF cycles are more likely to end in failure; this takes significant physical, emotional, and financial toll, from which 1 in 10 women never fully recover [22]. The likelihood of successful IVF procedure depends on numerous factors, many of which have been already defined and included in the available recommendations [23], but the variability in IVF success rate has not been fully explained yet [24].

Due to significant association of CT infection with female infertility [17,25], corresponding cervical screening has been strongly recommended as a prenatal test to be performed prior to initiating IVF, thus becoming a national standard of care in many countries [26]. However, the usefulness of serological and follicular fluid evidences of CT infection in predicting the outcome of IVF procedure has not been established yet. Therefore, the main objective of our study was to assess the potential association between serological and follicular fluid markers of CT infection and pregnancy or birth rates as indicators of IVF success.

## 2. Materials and Methods

### 2.1. Study Design and Participants

This prospective, observational, multicentre cohort study included female patients undergoing an IVF procedure at the Department of Medical Assistant Reproduction, University Clinical Centre Kragujevac, or at the Department of Artificial Reproductive Technology, The Obstetrics and Gynaecology Clinic “Narodni Front”, Belgrade, Serbia. Study participants were recruited based on convenient sampling from hospitalized patients. The main inclusion criteria aligned with the general eligibility requirements for IVF, as recommended by the European Society of Human Reproduction and Embryology (https://www.eshre.eu, accessed on 9 January 2019) and the Republic Fund of Health Insurance in Serbia (http://www.eng.rfzo.rs, accessed on 9 January 2019). In addition, study participants had to be free from active CT infection, as confirmed by negative polymerase chain reaction (PCR) analyses of endocervical and vaginal swabs. Patients younger than 18 years old, those already participating in another study, and those that were not willing to provide a written informed consent for participation were not included. The study conformed with the Declaration of Helsinki and the Good Clinical Practice, and was approved by the relevant Ethics Committee on 17 April 2019, decision No 01/19-1591.

### 2.2. Data Collection and Analyses

Prior to entering the study, all study participants signed written informed consent form. The IVF procedure at both study centers was performed according to the standard protocol [27]. Serum and follicular fluid samples were collected during the IVF procedure and stored at −20 °C for subsequent analysis.

IgG and IgA antibodies specific for CT major outer membrane protein (MOMP) antigen were determined in serum and follicular fluid using the EUROIMUN commercial ELISA test (Lubec, Germany). According to the manufacturer’s instructions, ELISA results for IgA were reported as a negative for signal-to-cutoff (S/CO) ratio lower than 0.8, borderline for values between 0.8 and 1.1, and positive for those higher than 1.1. Similarly, the results of IgG test were considered negative, borderline, or positive if the obtained values of relative units (RU/mL) were lower than 16, between 16 and 22, or above 22, respectively. In the present study, 5, 2, 2, and 1 patient, considered borderline for IgG in serum, IgG in follicular fluid, IgA in serum, and IgA in follicular fluid, respectively, were excluded from the analyses.

There were two primary outcomes in our study that served as major indicators of IVF success, as follows: (a) biochemical pregnancy, defined as the serum β-HCG, measured 14 days after embryo transfer, higher than 25U/L, and (b) live birth, i.e., giving birth to a living child or children after at least 28 weeks of pregnancy [28]. In addition, several other IVF parameters, including the number of oocytes retrieved and the number of embryos obtained, transferred, and implanted, were monitored, with the implantation rate per embryo transferred (EI/ET ratio) considered a secondary outcome in the study and also indicative of IVF success [29].

### 2.3. Statistical Analysis

Statistical analysis was performed with IBM SPSS Statistics, version 20 (IBM Inc, Armonk, NY, USA). Continuous data were tested for normality using the Kolmogorov–Smirnov test and presented as mean ± standard deviation (SD) or median with interquartile range (IQR), depending on the distribution. Categorical data were presented as count and percentage. The number of oocytes and embryos obtained and transferred in connection to IVF success was evaluated using Mann–Whitney U or χ^2^ test. The potential association of antibodies in serum and follicular fluid with indicators of IVF success, i.e., biochemical pregnancy, live birth rate, and EI/ET ratio, was tested using univariable logistic or ordinal regression. Potential overestimation due to the relatively small sample size was corrected by internal validation of the model using bootstrapping analysis with 1000 bootstrap resamples. Additional post hoc power analysis was performed using the Stata Statistical Software, release 16 (StataCorp LLC., College Station, TX, USA). The strength of the observed associations was presented as odds ratio (OR) with 95% confidence intervals, with the statistical significance level set at *p* < 0.05.

## 3. Results

There were 121 patients with a median age of 35 years (IQR: 32–39; range: 23–48) included in the study. After IVF procedure, at least one embryo was successfully implanted and biochemical pregnancy achieved in 55 (45.5%), with 51 of them (42.1% of the study population) giving birth to a living child or children. Women that achieved biochemical pregnancy had a significantly higher number of oocytes retrieved and number of embryos obtained (U = 1030.0, *p* < 0.001, and U = 704.5, *p* < 0.001, respectively), and the same was observed in those with successful delivery (U = 1083.0, *p* < 0.001, and U = 742.5, *p* < 0.001, respectively) (Table 1). Up to three embryos were transferred per patient during IVF, with no significant difference in their number between those with a successful and unsuccessful outcome, as assessed by both biochemical (χ^2(3)^ = 3.498, *p* = 0.321) and clinical indicators (χ^2(3)^ = 3.963, *p* = 0.266). The frequency of patients positive for IgG or IgA antibodies in serum or follicular fluid, as classified according to major IVF outcomes, is presented in Table 1.

The comparison between patients in terms of the IVF success rate revealed a significant association between older age and lower odds of achieving both biochemical pregnancy (Table 2) and live birth (Table 3). As expected, older patients were also less likely to have successful implantation per embryos transferred (Table 4). Regarding antibodies in serum or follicular fluid, a significant association with IVF outcome was not observed. Yet, there was a trend toward increased risk of IVF failure in patients positive for IgG in serum or positive for either IgG or IgA in follicular fluid, which further bootstrapping analyses indicated as statistically significant (Table 2 and Table 3). Based on the post hoc power analysis, the power of the study to demonstrate the true association of anti-MOMP antibodies in follicular fluid with IVF success rate was estimated to 0.923.

On the other hand, a significantly higher embryo implantation rate was detected among patients negative for antibodies in follicular fluid. Subsequent bootstrap resampling confirmed the observed association, and additionally suggested a positive IgG test in either serum or follicular fluid as predictive of a lower implantation rate per embryos transferred (Table 4).

## 4. Discussion

In the present study, detection of chlamydial anti-MOMP IgG and IgA in the serum and follicular fluid of infertile women demonstrated potential to be indicative of a lower IVF success rate, irrespective of maternal age. In the era of a dramatic decline in fertility rates worldwide [30], when IVF represents the most efficient assisted reproductive technology (ART) method [5,20], and sexually transmitted infections list among the most recent WHO global research priorities [31], we trust our findings to at least partly contribute to this extremely important, yet still unresolved topic [32]. In addition, we confirmed advanced maternal age to be associated with a higher risk of IVF failure, irrespective of CT infection. Witnessing a prominent societal shift toward delayed parenthood [33], with IVF featuring the possibility of oocyte donation [34], we believe our modest contribution to the further clarification of the importance of age for IVF outcome can help optimize personalized fertility solutions.

In an attempt to conceive and deliver, women with CT infection face a significant risk of failure, which calls for successful treatment as an important step in reaching the goal of child birth [35]. This could hold especially true in couples deemed infertile and preparing for IVF procedure, regardless of whether the infection represents the sole cause of infertility [36], or they also face other issues that hinder embryo implantation [5]. Unfortunately, not all forms of CT infections are easy to detect [37]; so, routine cervical screening often needs to be supplemented with other diagnostic methods to confirm or rule out the presence of this disease [38]. Among several options offered to date [39], CT antibody testing has been proposed as one of the most cost-effective screening methods for CT-associated genital tract pathology [40]. However, investigations of IVF outcome in relation to CT serological and follicular fluid markers yielded conflicting results [32,37].

The presence of CT-specific IgG and IgA antibodies in the follicular fluid of infertile women undergoing IVF was first detected 35 years ago by Lunenfeld et al. [41]. Failing to demonstrate the link between CT antibodies at the site of fertilization and fertilization rate, they proposed transudation of immunoglobulins from blood serum into ovarian follicles, rather than the presence of CT itself within the oocyte or embryo, as the most probable explanation for their finding. A subsequent similar study by Neuer et al. [42] confirmed the lack of association between IgG in follicular fluid and IVF outcome, but observed increased risk of failure after embryo transfer in women positive for follicular fluid CT anti-MOMP IgA, indicative of more recent or persistent form of disease [37,41,43]. While adhering to initial hypothesis of antibodies entering by transduction from the circulation, they introduced another possibility of local production of IgA in response to viable CT present and/or replicating in follicular fluid macrophages [42]. A later investigation by Pacchiarotti et al. [37], involving women entering IVF after antibiotic treatment of CT infection that resulted in negative cervical swab, showed that almost half of the patients remained positive for IgA in both serum and follicular fluids in spite of being clinically cured. What is more, IgA-positive women, as compared to those tested negative, had a significantly reduced number of mature oocytes, and half as high pregnancy and implantation rates. The authors hypothesized that the association between anti-CT antibodies and IVF outcome stems from a persistent immune response against both bacterial and host antigens that results in a low embryo quality, and suggests antibody detection rather than cervical swab test as a confirmation of CT treatment success [37]. On the other hand, in the study by Muller et al. [44] on patients diagnosed with TFI, despite the observed association between follicular fluid anti-CT IgG and IgA positivity and the severity of pelvic adhesions, women positive for follicular fluid antibodies did not significantly differ from others in terms of IVF success rate. In our study, the presence of IgG or IgA in follicular fluid showed clear association with worse implantation rate per embryos transferred, as well as the tendency to link with lower rates of biochemical pregnancy and live birth after IVF. Bearing in mind our relatively small sample size and relying on an association detected through approximation to a wider population, we are inclined to believe that the presence of anti-CT antibodies in follicular fluid can indicate a higher risk of an unfavorable IVF outcome, and thus, it could be considered useful to conduct additional diagnostic tests in patients undergoing laparoscopy due to mechanical infertility.

Yet, due to its invasiveness, the detection of anti-CT antibodies in follicular fluid has long been recognized as inappropriate for routine use [41], prompting search for other potential markers as a suitable alternative. As the earliest evidence of CT infection leading to tubal infertility were based on serological studies [45,46,47], the possibility that serum anti-CT antibodies could be linked to decreased fertilization rate has been extended to IVF research. The main focus was put on anti-MOMP [48] IgG and IgA, which have been described as indicative of past, and active or persistent CT infection, respectively [39,49,50]. However, the initial results were contradictory, ranging from complete lack of association between seropositivity and the outcome of IVF [46] to conclusions that the presence of IgG in serum decrease IVF success rate of IVF by half [49], or that the high prevalence of both IgG and IgA is associated with low chances of biochemical pregnancies achieved by IVF being ended in “bringing home a baby” [50]. While some of the subsequent studies conformed with the latter [37], others failed to detect any significant association [42], and the proposed link between CT seropositivity and lower IVF success rate remained unconfirmed [32,51]. In the present study, we failed to observe the association of seropositivity for IgG or IgA with live birth rate after IVF. Yet, we noticed a tendency of women positive for serum IgG antibody to display a lower implantation rate per embryo transferred and be less likely to achieve biochemical pregnancy, further supported by predictions drawn from larger dataset obtained by resampling from our population. Although our study lacks clear-cut conclusion in terms of IVF outcome, our results align with the most frequent hypothesis of CT infection affecting fertilization success by hindering embryo implantation, either due to the endometrium being less receptive, or due to cross-reactivity between chlamydial and human antigens triggering an autoimmune response [17,44].

Since MOMP represents the most predominant and structurally stable surface-exposed CT protein that elicits strong IgG and IgA immune responses [52], in the present study, it was considered a valid and well-established serological marker of CT infection. However, this type of testing is far from ideal, mostly because of false positive and false negative results, low negative predictive value, as well as the lack of scientific consensus on which phase of CT infection course over time the positive test results actually reflect [53,54,55,56,57]. Yet, being simple and safe, serological testing can complement patient selection and preparation for IVF by initial screening for indications for other more invasive diagnostic procedures [35,40]. This could be especially relevant for persistent CT infection: the constant presence of pathogen and the sustained immune response enable long-term maintenance of high antibody concentrations, therefore proposing a serum-based assay as the most desirable for its diagnosis [58]. In addition, previous reports of other CT-related antibodies associated with tubal pathology and/or affecting IVF pregnancy rate [54,56,59,60] introduce a possibility of several serological markers being integrated into a serological panel, which would be predictive of unfavorable IVF outcome with greater sensitivity and specificity [39,61]. One combination of individual CT IgG antibodies (MOMP, TARP, CPAF, OMP2, and HSP60) has already demonstrated advanced clinical predictive value in regard to TFI [61], further supporting the role of serological panel implementation in predicting IVF success rate.

On the other hand, the importance of maternal age for childbearing in general has always been considered a common knowledge [62]. Following introduction of ART, the available data supported apparent expectations, and the advanced age of a mother entering the procedure was deemed the most significant negative factor affecting IVF outcome [63]. However, when controlling for other relevant variables, it was revealed that maternal age is associated with live birth rates and not with embryo implantation [64], suggesting that the age of the oocytes, rather than the age of a bearer, represent the true obstacle in achieving pregnancy in otherwise healthy mothers [65]. The delay of childbearing that became omnipresent during the past several decades [66] thus welcomed oocyte donation as a promising solution for women with “biological clock” ticking [67,68]. Unfortunately, even with the help of a donor, the risk of adverse pregnancy outcomes in women older than 45 years remains controversial [69,70]. In the present study, where the oldest participant was 48 years old and all the oocytes used were autologous, advanced maternal age was associated with a significantly lower implantation rate per embryo transferred, and lower odds of both achieving biochemical pregnancy and giving birth to a living child or children. The effect we observed was independent of CT infection and its consequences. Almost half a century after the first oocyte donation took place [71], we believe advanced maternal age in relation to IVF still deserves the attention of researchers and hope our observations, together with future studies, will help improve IVF success rates.

Our study suffers from several important limitations. First, the study utilized single-antigen-based serological testing, which displays lower sensitivity and specificity compared to previously reported serological panels [61]. In addition, the test we employed may have been based on a single serovar, which could introduce a potential bias in antibody detection. Second, even though the estimated power of the study indicates high probability that the observed association between anti-MOMP antibodies and IVF success rate truly exists in the population, our small sample size precludes the ability of the power calculation to consider the effect of confounders. Last but not least, our study lacks data on other significant factors that could (or have been shown to) affect IVF success rate, including embryo and sperm quality, underlying health conditions, or hormonal levels [23]. Moreover, it has been observed that semen anti-MOMP antibodies are significantly associated with TFI in female partners, indicating a possible transmission of immunogenic factors that could impair fertilization [72]. Therefore, the additional screening of male partners for anti-MOMP antibodies, which we were not able to perform, would provide additional information regarding couple-level immunological interactions influencing IVF outcome.

In conclusion, our results support the potential role of serum and follicular fluid IgG and IgA antibodies, and confirm the importance of maternal age, in predicting the outcome of IVF. Considering the study limitations, we encourage other researchers to challenge our results by additional investigations.

## Figures and Tables

**Table 1:** Age, IVF parameters, and CT antibodies in subjects classified according to major indicators of IVF success.

		Biochemical Pregnancy		Live Birth	
		Yes	No	Yes	No
Age ^1^		34.0 (30.0–37.3)	36.0 (33.0–40.0)	34.0 (30.0–36.3)	36.0 (33.0–40.0)
Number of oocytes ^1^	Retrieved	9 (7–12)	6 (3–9)	9 (7–12)	7 (3–9)
	Obtained	6 (4–8)	3 (2–4)	6 (4–8)	3 (2–4)
	Transferred	2 (1–2)	2 (2–2)	2 (1–2)	2 (2–2)
	Implanted	1 (1–1)	0 (0–0)	1 (1–1)	0 (0–0)
IgG ^2^	Serum	3 (5.7%)	11 (17.5%)	3 (6.1%)	11 (16.4%)
	Follicular fluid	2 (3.7%)	8 (12.3%)	2 (4.0%)	8 (11.6%)
IgA ^2^	Serum	4 (7.4%)	8 (12.3%)	4 (7.8%)	8 (11.8%)
	Follicular fluid	1 (2.0%)	3 (4.8%)	1 (2.1%)	3 (4.5%)

^1^ Presented as median (IQR); ^2^ presented as number (frequency) of subjects.

**Table 2:** Summary of variable estimates using univariable logistic regression analysis related to biochemical pregnancy after IVF.

		Univariable Logistic Regression Analysis						Bootstrapping Analysis			
Variables		B	SE	Wald χ^2^	*p*	OR	95% CI ^1^	B	SE	*p*	95% CI ^2^
Age		−0.117	0.043	7.284	0.007	0.890	0.817; 0.969	−0.117	0.042	0.005	−0.210; −0.043
IgG	Serum	−1.260	0.681	3.426	0.064	0.284	0.075; 1.077	−1.260	4.188	0.037	−21.202; 0.021
	Follicular fluid	−1.294	0.813	2.532	0.112	0.274	0.056; 1.350	−1.294	6.688	0.062	−21.293; 0.092
IgA	Serum	−0.562	0.642	0.766	0.381	0.570	0.162; 2.007	−0.562	2.807	0.385	−2.416; 0.765
	Follicular fluid	−0.933	1.171	0.635	0.425	0.393	0.040; 3.901	−0.933	11.505	0.175	−21.329; 21.336
IgG or IgA	Serum	−0.874	0.564	2.403	0.121	0.417	0.138; 1.260	−0.874	1.563	0.114	−2.628; 0.160
	Follicular fluid	−1.550	0.800	3.756	0.053	0.212	0.044; 1.018	−1.550	6.442	0.038	−21.263; −0.215

B—the regression coefficient or estimate; SE—standard error; Wald χ^2^—Wald test statistics for the degree of freedom of 1 (df = 1); *p*—probability; OR—odds ratio, calculated as exponent of B; 95%CI—95% confidence interval; ^1^—95% CI for the estimated OR; ^2^—percentile 95% CI for the estimated B; negative antibody tests were used as a reference category.

**Table 3:** Summary of variable estimates using univariable logistic regression analysis related to live birth after IVF.

		Univariable Logistic Regression Analysis						Bootstrapping Analysis			
Variables		B	SE	Wald χ^2^	*p*	OR	95% CI ^1^	B	SE	*p*	95% CI ^2^
Age		−0.099	0.043	5.327	0.021	0.906	0.833; 0.985	−0.099	0.042	0.010	−0.193; −0.2029
IgG	Serum	−1.103	0.681	2.621	0.105	0.332	0.087; 1.261	−1.103	4.081	0.057	−20.958; 0.048
	Follicular fluid	−1.147	0.814	1.985	0.159	0.318	0.064; 1.566	−1.147	6.810	0.095	−21.079; 0.314
IgA	Serum	−0.449	0.643	0.488	0.485	0.638	0.181; 2.249	−0.449	2.806	0.491	−2.219; 0.838
	Follicular fluid	−0.784	1.171	0.448	0.503	0.457	0.046; 4.530	−0.784	10.930	0.239	−21.131; 21.389
IgG or IgA	Serum	−0.751	0.564	1.771	0.183	0.472	0.156; 1.426	−0.751	2.003	0.159	−2.459; 0.229
	Follicular fluid	−1.391	0.800	3.022	0.082	0.249	0.052; 1.194	−1.391	6.866	0.049	−21.146; −0.025

B—the regression coefficient or estimate; SE—standard error; Wald χ^2^—Wald test statistics for the degree of freedom of 1 (df = 1); *p*—probability; OR—odds ratio, calculated as exponent of B; 95% CI—95% confidence interval; ^1^—95% CI for the estimated OR; ^2^—percentile 95% CI for the estimated B; negative antibody tests were used as a reference category.

**Table 4:** Summary of variable estimates using ordinal regression analysis related to EI/ET ratio after IVF.

		Univariable Logistic Regression Analysis						Bootstrapping Analysis		
Variables		B	SE	Wald χ^2^	*p*	OR	95% CI ^1^	B	SE	95% CI ^2^
Age		−0.118	0.041	8.495	0.004	0.888	0.820; 0.962	−0.118	0.040	−0.211; −0.055
IgG	Serum	1.308	0.681	3.688	0.055	3.698	0.973; 14.053	1.308	3.625	0.239; 18.053
	Follicular fluid	1.431	0.836	9.926	0.087	4.181	0.812; 21.535	1.431	5.426	0.327; 18.073
IgA	Serum	0.688	0.640	1.157	0.282	1.990	0.568; 6.977	0.688	2.520	−0.326; 2.525
	Follicular fluid	1.186	1.237	0.920	0.337	3.275	0.290; 36.991	1.186	7.558	−0.274; 17.107
IgG or IgA	Serum	0.909	0.557	2.663	0.103	2.481	0.833; 7.391	0.909	1.148	−0.055; 2.594
	Follicular fluid	1.659	0.819	4.104	0.043	5.254	1.055; 26.152	1.659	5.312	0.522; 18.124

EI/ET ratio—the implantation rate per embryos transferred; B—the regression coefficient or estimate; SE—standard error; Wald χ^2^—Wald test statistics for the degree of freedom of 1 (df = 1); *p*–probability; OR—odds ratio, calculated as exponent of B; 95%CI—95% confidence interval; ^1^—95% CI for the estimated OR; ^2^—percentile 95% CI for the estimated B; positive antibody tests were used as a reference category.

## Data Availability

The data presented in this study are available upon request from the corresponding author.

## Abbreviations

The following abbreviations are used in this manuscript:

ART	Assisted reproductive technology
B	Regression coefficient or estimate
CT	*Chlamydia trachomatis*
Df	Degree of freedom
ELISA	Enzyme-Linked Immunosorbent Assay
IQR	Interquartile range
IVF	In vitro fertilization
MOMP	Major outer membrane protein
OR	Odds ratio
P	Probability
PCR	Polymerase chain reaction
PID	Pelvic inflammatory disease
RU	Relative unit
S/CO	Signal-to-cutoff
SD	Standard deviation
SE	Standard error
TFI	Tubal factor infertility
Wald χ^2^	Wald test statistics for df = 1
